# Co-delivery of PD-L1- and EGFR-targeting siRNAs by synthetic PEG_12_-KL4 peptide to the lungs as potential strategy against non-small cell lung cancer

**DOI:** 10.1016/j.ejpb.2024.114177

**Published:** 2024-02

**Authors:** Rico C.H. Man, Yingshan Qiu, Susan W.S. Leung, Gilbert O. Fruhwirth, Jenny K.W. Lam

**Affiliations:** a Department of Pharmacology and Pharmacy, LKS Faculty of Medicine, The University of Hong Kong, 21 Sassoon Road, Hong Kong SAR; b Imaging Therapies and Cancer Group, Comprehensive Cancer Centre, School of Cancer and Pharmaceutical Sciences, King’s College London, London SE1 9RT, UK; c Department of Pharmaceutics, UCL School of Pharmacy, University College London, 29-39 Brunswick Square, London WC1N 1AX, UK

**Keywords:** Immune checkpoints, Lung cancer, Peptide-based vectors, Pulmonary delivery, RNA interference, siRNA delivery

## Abstract

**Background:**

Small interfering RNA (siRNA) holds great promise for treating various lung diseases, but the lack of safe and efficient pulmonary siRNA delivery systems has hindered its advance into the clinics. The epidermal growth factor receptor (EGFR) which promotes cell proliferation, and the programmed cell death ligand 1 (PD-L1) which plays a crucial role in suppressing cytotoxic T cells activity, are two important targets for treating non-small cell lung cancer (NSCLC). Here, we explored the potential of PEG_12_-KL4, a synthetic peptide, to deliver siRNA to various NSCLC cells and to lung tissues in mice.

**Methods:**

PEG_12_-KL4 was used to transfect siRNAs targeted at both EGFR and PD-L1 into NSCLC cells. Immunoblotting was used to evaluate the siRNA silencing effects in HCC827 and NCI-H1975 NSCLC cells. CD8+ T cell-mediated NSCLC cell killing was employed to demonstrate the functional effects of PD-L1 siRNA knock-down. Fluorescent siRNAs were used to visualise siRNA uptake in cells as well as to enable biodistribution studies in BALB/c mice.

**Results:**

Our results showed that PEG_12_-KL4 was efficient in mediating siRNA knock-down of EGFR and PD-L1 in various NSCLC cells. Importantly, the PEG_12_-KL4 peptide enabled significantly better siRNA delivery than the commercial Lipofectamine 2000 reagent. We hypothesised that PEG_12_-KL4 peptide enabled siRNA to either escape from or bypass endosomal degradation as indicated by confocal fluorescence imaging. Notably, combined knock-down of EGFR and PD-L1 in NCI-H1975 cells resulted in better effector T cell-mediated cancer cell killing than knock-down of PD-L1 alone. Moreover, biodistribution of PEG_12_-KL4/siRNA complexes following intravenous administration revealed poor lung delivery with the fluorescent siRNA accumulating in the liver. In contrast, intratracheal delivery of PEG_12_-KL4/siRNA complexes resulted in the fluorescent siRNA to be detected in the lung with retarded renal excretion.

**Conclusion:**

In conclusion, we demonstrated that the co-delivery of siRNAs targeting EGFR and PD-L1 using PEG_12_-KL4 is feasible and represents a promising future strategy to treat NSCLC, whereby pulmonary siRNA delivery is favourable to intravenous administration.

## Graphical abstract

**Figure:**
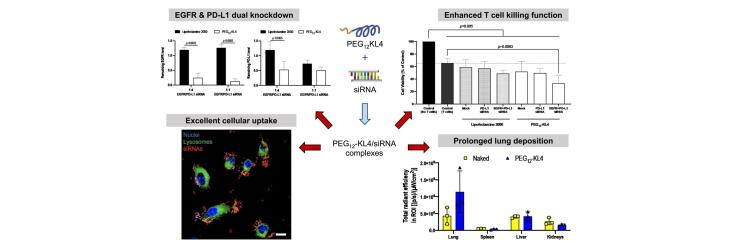

## 1 Introduction

Lung cancer is one of the leading causes of mortality worldwide. It is the most common cancer and the most common causes of cancer death, contributed to around 1.80 million deaths in 2020 [1], placing a huge burden on the healthcare system. Besides, lung cancer is frequently metastatic, rendering it extremely difficult to treat. Among all sub-types of lung cancer, non-small-cell lung cancer (NSCLC) is the most prevalent, accounting for 80–85 % of lung cancer diagnosis [2]. Chemotherapy has been the major drug-based therapy against NSCLC, but it lacks specificity and is associated with severe systemic side effects. Despite the modest survival benefit, more than half of the patients still relapse [3]. New and effective therapeutics against NSCLC are still highly sought-after.

In recent years, the major advances in the field of NSCLC treatment are the development of epidermal growth factor receptor (EGFR) tyrosine kinase inhibitors (TKIs) and immune checkpoint inhibitors targeting programmed cell death-1 receptor (PD-1)/ligand (PD-L1) [4], [5]. NSCLC is strongly associated with the over-expression of EGFR. Upon activation of EGFRs, the intracellular tyrosine kinase domain is phosphorylated and initiates a series of downstream signalling, resulting in cell survival and proliferation [6]. In consequence, EGFR has become an attractive target to combat NSCLC. EGFR-TKIs are clinically proven as effective anticancer agents. However, acquired resistance to EGFR-TKIs frequently develops within 12 months [7]. On the other hand, immune checkpoints are important regulators which prevent the immune system from indiscriminately attacking normal cells. Cancer cells can evade immune surveillance by stimulating immune checkpoint targets. PD-1/PD-L1 are one of the most widely studied immune checkpoints, and the expression of PD-L1 is significantly upregulated in NSCLC [8], [9]. PD-L1 presents on the surface of cancer cells and binds to PD-1 which presents on the surface of T cells. This interaction promotes the immune evasion of cancer cells through inhibiting the activity of cytotoxic T cells. Antibody-based PD-1/PD-L1 immune checkpoint inhibitors, which block the activation of PD-1/PD-L1 pathway, result in survival benefits for patient with advanced NSCLC [10]. However, only a subset of patients benefits from PD-1/PD-L1 inhibitor monotherapy [11].

RNA interference (RNAi) through small interfering RNA (siRNA) is a promising therapeutic platform for many diseases including cancer [12], [13]. It achieves gene silencing at post-transcriptional level in a sequence-specific manner. siRNA works through binding to the target mRNA and mediates mRNA degradation, thereby inhibiting gene expression. Compared to small drug molecules (EGFR-TKIs) and monoclonal antibodies (PD-1/PD-L1 checkpoint inhibitors), the two most important types of drug molecules in NSCLC therapy, siRNA has several distinct advantages. siRNA performs its function through the complementary binding to the target mRNA, whereas small molecules and monoclonal antibody drugs need to recognise and interact with the complex spatial conformation of the target proteins. In theory, siRNA is able to target any gene of interest including those that are not ‘druggable’ by convention drug molecules or antibodies. Delivery of several drug molecules and/or antibodies are not always feasible and can potentially increase toxic side effects [14]. In contrast, it is possible to deliver various siRNA sequences to the same tumour site to silence multiple genes simultaneously and this approach holds great promise for effective NSCLC treatment.

This study investigated the co-delivery of siRNAs targeting EGFR and PD-L1 to human lung cancer cells. Previously, our group has demonstrated the feasibility of employing the synthetic PEG_12_-KL4 peptide to deliver RNAs to lung epithelial cells by using reporter (luciferase) or housekeeping genes (glyceraldehyde 3-phosphate dehydrogenase) [15], [16]. The PEG_12_-KL4 consists of surfactant peptide KL4 and monodisperse PEG with 12 repeating units. KL4 peptide, which was originally developed to mimic surfactant protein B to reduce surface tension at the air/liquid interface in the lung [17], showed to be an effective RNA transfection agent [18], [19]. PEGylation can enhance the solubility and improve the safety profile of KL4 peptide following pulmonary delivery in healthy mice [15]. Here, we further explored the therapeutic application of this peptide to deliver siRNAs targeting EGFR and PD-L1 to lung cancer cells.

## 2 Materials and Methods

### 2.1 Materials and reagents

PEG_12_-KL4 peptide (sequence: PEG_12_-KLLLLKLLLLKLLLLKLLLLK-NH_2_) was purchased from EZBiolab (Carmel, NJ, USA) with the purity over 90 %. The peptide stock solution was prepared in ultrapure water at 1 mg/mL and was stored at 4 °C. EGFR siRNAs were purchased from Integrated DNA Technologies (San Diego, CA, USA). ON-TARGETplus™ Human PD-L1 siRNA and siGLO Cyclophilin B Control siRNA (cyanine 3 fluorescently labelled siRNA) were purchased from GE Healthcare Dharmacon (Lafayette, CO, USA). The Silencer® Select Negative Control siRNA was purchased from ThermoFisher Scientific (Waltham, MA, USA). The information of siRNAs is summarised in Table S1 (Supplementary Information). All siRNAs were stored at −20 °C as powder or reconstituted siRNAs solutions. All cell culture media and reagents (except for culturing T cells), Western immunoblot reagents, Lipofectamine 2000, Hoechst 33342 stain and Lysotracker Green DND-26 were purchased from ThermoFisher Scientific (Waltham, MA, USA). T cell culture media and reagents were purchased from STEMCELL Technologies (Cambridge, UK). Anti-EGFR and anti-PD-L1 antibodies were purchased from Cell Signalling Technology (Danvers, MA, USA). Anti-β-actin antibody was purchased from Abcam (Cambridge, UK). Secondary antibodies were purchased from Jackson ImmunoResearch (West Grove, PA, USA). Other reagents were obtained from Sigma-Aldrich (Saint Louis, MO, USA) as analytical grade or better.

### 2.2 Cell culture

BEAS-2B cells (human bronchial epithelial cells), A549 cells (human alveolar epithelial adenocarcinoma), NCI-H292 cells (human lung mucoepidermoid carcinoma), HCC827 cells (human lung epithelial adenocarcinoma), and NCI-H1975 cells (human lung epithelial adenocarcinoma) were purchased from American Type Culture Collection (ATCC; Manassas, VA, USA). The human peripheral blood mononuclear cells (PBMCs) were purchased from STEMCELL Technologies (Cambridge, UK). BEAS-2B cells were cultured in Keratinocyte-SFM (serum-free medium) supplemented with human recombinant Epidermal Growth Factor (rEGF), Bovine Pituitary Extract (BPE) and 100 IU/mL penicillin/streptomycin (P/S). A549 cells were cultured in DMEM supplemented with 10 % (v/v) heat-inactivated fetal bovine serum (FBS) and 100 IU/mL P/S. NCI-H292 cells, HCC827 cells and NCI-H1975 cells were cultured in RPMI 1640 supplemented with 10 % (v/v) heat-inactivated FBS, 100 IU/mL P/S, and 2 mM L-glutamine. All the lung cells were maintained in a humidified incubator at 37 °C/5% CO_2_. Monthly mycoplasma testing was performed using LookOut® Mycoplasma PCR Detection Kit (Sigma-Aldrich) and all cell lines found to be mycoplasma-negative throughout experimentation.

### 2.3 siRNA transfection study

PEG_12_-KL4/siRNA complexes were prepared at 10:1 ratio (w/w). Lipofectamine 2000/siRNA complexes were prepared at 2:1 ratio (w/w). All complexes were prepared in OptiMEM I reduced serum media. The complexes were mixed thoroughly with a vortex shaker and incubated at room temperature for at least 30 min. NCI-H292, HCC827, NCI-H1975 cells were seeded in six-well plates at a density of 2 × 10^5^ cells per well overnight. The cells were transfected with siRNAs targeting EGFR and/or PD-L1 as PEG_12_-KL4/siRNA complexes or Lipofectamine 2000/siRNA complexes. After 5 h of incubation, the transfection media was removed. The cells were washed once with PBS and replaced with normal culturing media. At 48 h post-transfection, the cells were washed and scraped in 100 μL RIPA lysis buffer supplemented with protease inhibitor cocktail. The cell lysates were collected, incubated on ice for 30 min, and then centrifuged at 15000 rpm for 5 min. The supernatant was collected and stored at −20 °C for Western blot analysis to evaluate the expressions of target proteins.

### 2.4 Western blot analysis

Cell lysates containing 20 μg of protein were loaded into 10 % sodium dodecyl sulfonate (SDS)-polyacrylamide gel. Following electrophoresis, the proteins were transferred to PVDF Immobilon-P membranes (EMD Millipore), which were blocked in 5 % BSA (w/v) for 1 h. After washing, the membranes were incubated with primary antibodies overnight at 4˚C on shaking. The membranes were washed again and incubated with the corresponding secondary antibody conjugated to horseradish peroxidase for 1 h at room temperature. Signals were detected using an enhanced chemiluminescence substrate (ECL, Thermo) according to the manufacturer’s instructions with a gel documentation system (Syngene Chemi G:Box XX6 Model). EGFR and PD-L1 expression was analysed by densitometry using ImageJ software (Version 1.8.0, NIH, USA). The remaining EGFR or PD-L1 expression was the density of the EGFR or PD-L1 band of samples transfected with positive siRNA (normalised with β-actin band of the corresponding samples) divided by the EGFR or PD-L1 band of the samples transfected with the negative siRNA (normalised with β-actin band of the corresponding samples).

### 2.5 Cellular uptake study by live cell confocal microscopy

Confocal microscopy was performed to study the cellular uptake of fluorescent siRNAs mediated by PEG_12_-KL4 or Lipofectamine 2000 in NCI-H1975 cells. The cells were plated in a 35 mm Mattek glass bottom culture dish (Mattek Corp. Ashland, MA, USA) at a seeding density of 2 × 10^5^ cells per dish overnight. The cells were transfected with naked siRNA, Lipofectamine 2000/siRNA complexes or PEG_12_-KL4/siRNA complexes containing 2 µg of fluorescently labelled siRNA. After 4 h of incubation, the transfection media containing the siRNA complexes was removed, and the cells were washed. Subsequently, Hoechst stain prepared at a concentration of 3 μg/mL was added to the cells for nuclei staining. After 30 min, the cells were washed to remove the Hoechst stain. Lysotracker at a concentration of 50 nM was added to the cells for lysosomes staining. The cells were imaged by an inverted laser scanning confocal microscope (Zeiss LSM 780 inverted microscope, Jena, Germany) with lasers appropriate for imaging Hoechst (diode laser ex 405 nm), AlexaFluor-488 (argon laser ex 488 nm) and Cy-3 (diode-pumped solid state laser ex 561 nm) coupled with two photomultiplier tube (PMT) detectors, one 32-channel spectral gallium arsenide phosphide (GaAsP) detector, and one transmitted light PMT detector.

### 2.6 Isolation and characterisation of human CD8+ T cells

Human CD8+ T cells were isolated from human PBMCs using EasySep™ Human CD8+ T Cell Enrichment Kit (STEMCELL Technologies, Cambridge, UK). The isolated human CD8+ T cells were cultured in ImmunoCult™-XF T Cell Expansion Medium with 10 ng/mL of human recombinant IL-2 and activated by 25 µL/mL of ImmunoCult™ Human CD3/CD28/ CD2 T Cell Activator for 3 days with growth media replenishment every 2–3 days. The PBMCs and isolated CD8+ T cells were characterised by staining with monoclonal mouse anti-human CD3 antibody (STEMCELL Technologies; clone OKT3) conjugated with FITC and monoclonal mouse anti-human CD8a antibody (STEMCELL Technologies; clone SK1) conjugated with APC for 30 min in the dark. The cells were washed three times with PBS and analysed by a flow cytometer (BD FACSCantoII Analyser, BD Biosciences, CA, USA). Data were subsequently analysed by FlowJo software (Version 10.4).

### 2.7 Human CD8+ T cell functionality study

NCI-H1975 cells were seeded in 24-well plates at a density of 1 × 10^5^ cells per well overnight. To identify the optimal effector: target ratio for cancer cell killing, isolated human CD8+ T cells (effector) were co-cultured with cancer cells (target) in a range of ratios for 24 h. The cells were washed with PBS five times to remove the CD8+ T cells and 200 μL of MTT solution (0.8 mg/mL) was added to each well. After 2 h of incubation, the MTT solution was removed and 200 μL of isopropanol was added to dissolve the violet formazan crystals. The absorbance at 570 nm was measured by a microplate reader (Multiskan GO, Thermo scientific, Massachusetts, USA). Next, NCI-H1975 cells were transfected with siRNAs targeting PD-L1 alone or both EGFR and PD-L1 (1:1 ratio) with a total concentration of 100 nM as PEG_12_-KL4/siRNA complexes for 48 h, followed by co-culture with human CD8+ T cells at indicated effector: target ratio for additional 24 h. The cellular viability was measured by MTT assay.

### 2.8 Animals

Young adult female BALB/c mice (7–9 weeks old and with body weights ranging 18–22 g) were used in all experiments. All mice were housed within The Centre for Comparative Medicine Research of The University of Hong Kong under specific pathogen-free conditions in a dedicated and licensed air-conditioned animal room (at 23 ± 2 °C and 40–60 % relative humidity) under light/dark cycles lasting 12 h every day. All procedures were approved by the Committee on the Use of Live Animals for Teaching and Research (CULATR) at the University of Hong Kong (CULATR number 4931–19).

### 2.9 *In vivo* siRNA biodistribution

PEG_12_-KL4/siRNA complexes prepared at ratio 10:1 (w/w) containing 10 μg of fluorescently labelled siRNA were intratracheally administered (in 75 µL PBS) by MicroSprayer Aerosoliser (PennCentury Inc., PA, USA) or intravenously administered (in 200 µL PBS) to the mice. The administration procedure was previously described [15]. Naked siRNA (10 μg per mouse) was included as controls for comparison. At indicated timepoints, the mice were sacrificed by intraperitoneal injection with a lethal dose of pentobarbital (150 mg/kg). The lung, liver, kidneys and spleen were harvested. The spleen served as the background control for signal adjustment. Fluorescence imaging of the harvested organs was performed with an IVIS® Spectrum *In vivo* imaging system (PerkinElmer, USA) equipped with excitation and emission wavelength bandpass filters of 535 ± 15 nm and 580 ± 10 nm, respectively. Regions of interest (ROI) were manually drawn surrounding the harvested organs and used to calculate the radiant efficiency.

### 2.10 Statistical analysis

Prism software version 9 (GraphPad, La Jolla, USA) was used to calculate all statistical parameters as indicated. For all cell culture experiments, the data were analysed by one-way or two-way ANOVA followed by Tukey’s post-hoc test. For the biodistribution study, the data were analysed by Student’s *t*-test to compare the biodistribution of fluorescent siRNAs in the organs with or without PEG_12_-KL4. Generally, *p*-values were calculated using significance levels of α = 0.05. All experiments were repeated at least three times independently unless otherwise indicated. Differences were considered as statistically significant at *p* < 0.05.

## 3 Results and discussion

### 3.1 Expression of EGFR and PD-L1 in human NSCLC cell lines

**Fig. 1:**
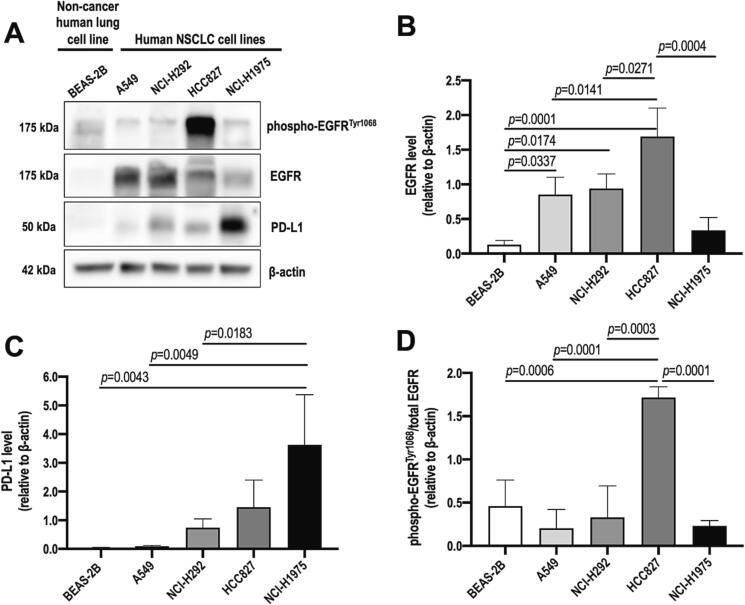
The baseline expressions of EGFR and PD-L1 in human lung cell lines. (A) Western blot images showing the cell expressions of EGFR and PD-L, with β-actin used as internal control. Densitometry analysis was presented as the ratio of the band density of (B) EGFR and (C) PD-L1 to that of β-actin. (D) The ratio of phosphorylated-EGFR^Tyr1068^/total EGFR (both normalised to β-actin) was calculated. The data were shown as mean ± SD of three independent replicates (n = 3) and were compared by one-way ANOVA followed by Tukey’s post-hoc test.

The baseline expression levels of EGFR and PD-L1 were first evaluated in a panel of human NSCLC cell lines, including A549, NCI-H292, HCC827 and NCI-H1975 cells. In addition, non-cancerous human bronchial epithelial cells, BEAS-2B cells, were included as a negative control. Immunoblot analysis showed that the expression of EGFR and PD-L1 were up-regulated in NSCLC cell lines but not in healthy airway epithelial cells, demonstrating the correlation between NSCLC and elevated EGFR / PD-L1 levels (Fig. 1A-C). Previous studies also reported that NSCLC is strongly associated with high levels of EGFR and PD-L1, leading to poor patient prognosis [20], [21]. Among the four NSCLC cell lines tested, A549 and NCI-H292 cells present wild-type EGFR status whereas HCC827 and NCI-H1975 cells display mutant EGFR status [22]. HCC827 cells harbour an in-frame exon 19 deletion in the EGFR tyrosine kinase domain (ΔE746-A750 deletion, exon 19) [23], whereas NCI-H1975 cells harbour L834R/T766M double mutations in EGFR [24]. It was previously reported that the PD-L1 protein level in NSCLC cells with mutant EGFR was significantly higher than that in cells with wild-type EGFR [20]. The study also suggested that this PD-L1 up-regulation was attributed to the activation of ERK1/2/c-Jun signalling pathways. Here, NCI-H1975 cells demonstrated the highest PD-L1 expression, followed by HCC827 cells (Fig. 1C), corroborating that EGFR-mutant cells are likely to express higher PD-L1 levels. In addition, HCC827 exhibited a higher degree of EGFR phosphorylation (Fig. 1D), possibly due to the acquired EGFR mutation of exon 19 E722-A726 deletion resulting in continuous EGFR autophosphorylation. Collectively, as NCI-H292, HCC827 and NCI-H1975 cells showed relatively high baseline expressions of both EGFR and PD-L1, they were chosen for subsequent siRNA transfection study.

### 3.2 Individual transfection of siRNA targeting EGFR and PD-L1

**Fig. 2:**
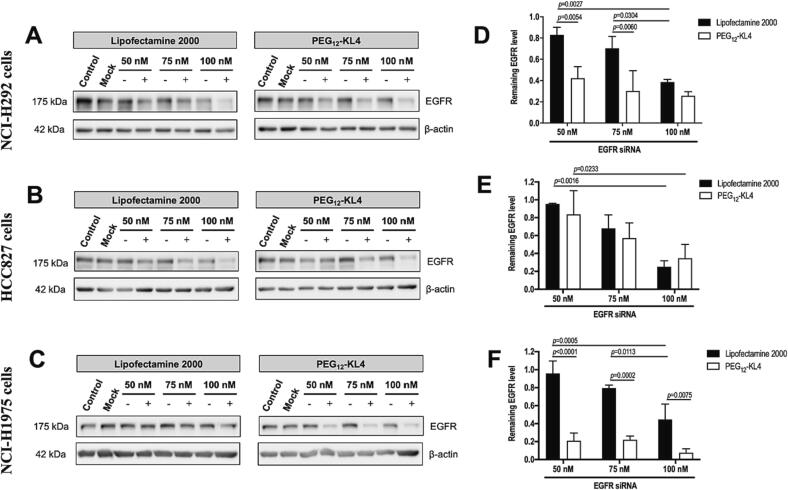
Transfection of siRNA targeting EGFR on human NSCLC cells. The cells were transfected with EGFR siRNA (+) or negative control siRNA (-) complexes prepared with PEG_12_-KL4 at 10:1 (w/w) or Lipofectamine 2000/siRNA at 2:1 ratio (w/w). Untreated cells and cells treated with delivery vector only (mock) were included. (A-C) Representative Western blot of EGFR expression at 48 h post-siRNA transfection with β-actin as internal control. (D-F) Densitometry analysis were illustrated as the mean ± SD of three independent replicates (n = 3). The data were analysed by two-way ANOVA followed by Tukey’s post-hoc test.

The transfection of siRNA targeting EGFR was performed on NCI-H292, HCC827 and NCI-H1975 cells at three siRNA concentrations (50, 75 and 100 nM). EGFR protein expression was evaluated by Western blot 48 h after transfection (Fig. 2). In NCI-H292 cells, the expression of EGFR protein was reduced to levels below 40 % of the control cells (transfected with negative control siRNA) when using 100 nM of siRNA (Fig. 2A and D). PEG_12_-KL4 improved EGFR knock-down effect compared to Lipofectamine 2000, but no significant difference was observed among the three tested concentrations when the peptide was used to transfect siRNA. In HCC827 cells, the siRNA transfection efficiencies of PEG_12_-KL4 and Lipofectamine 2000 were also similar (Fig. 2B and E). The highest knock-down effect was achieved at 100 nM of siRNA, in which the EGFR protein levels were reduced to < 40 % compared to the control cells. PEG_12_-KL4 outperformed Lipofectamine 2000 as a transfection agent for siRNA-mediated EGFR protein knock-down in NCI-H1975 cells (10 to 20 % residual EGFR protein when using the peptide, while 50 % when using Lipofectamine 2000; Fig. 2C and F). Overall, PEG_12_-KL4 was efficient in mediating siRNA knock-down of EGFR in all three lung cancer cell lines. The peptide also performed better than Lipofectamine 2000 in cells that are hard to transfect such as NCI-H1975.

**Fig. 3:**
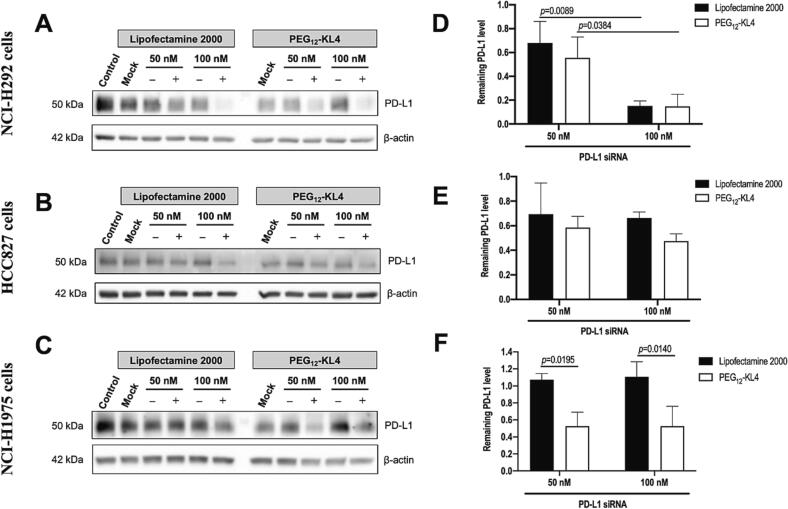
Transfection of siRNA targeting PD-L1 on human NSCLC cells. The cells were transfected with PD-L1 siRNA (+) or negative control siRNA (-) complexes prepared with PEG_12_-KL4 at 10:1 (w/w) or Lipofectamine 2000/siRNA at 2:1 ratio (w/w). Untreated cells and cells treated with delivery vector only (mock) were included. (A-C) Representative Western blot of PD-L1 expression at 48 h post-siRNA transfection with β-actin as internal control. (D-F) Densitometry analysis were illustrated as the mean ± SD of three independent replicates (n = 3). The data were analysed by two-way ANOVA followed by Tukey’s post-hoc test.

Transfection of siRNA targeting PD-L1 was performed on the same NSCLC cell lines as above at two siRNA concentrations (50 and 100 nM), and PD-L1 protein expression was examined by immunoblot analysis 48 h after transfection (Fig. 3). In NCI-H292 cells, the PD-L1 knock-down effect was similar between PEG_12_-KL4 and Lipofectamine 2000 (Fig. 3A and D), and the level of PD-L1 expression was strongly dependent on siRNA concentrations. The PD-L1 level was reduced from above 50 % at 50 nM of siRNA, to below 20 % when the siRNA concentration was increased to 100 nM. In HCC827 cells, the PD-L1 knock-down was less satisfactory, with PD-L1 level remained above 50 % at 100 nM of siRNA for both PEG_12_-KL4 and Lipofectamine 2000 (Fig. 3B and E). In NCI-H1975 cells, PD-L1 knock-down was observed only when the cells were transfected with PEG_12_-KL4 but not with Lipofectamine 2000 (Fig. 3C and F). The transfection efficiency of PEG_12_-KL4 was similar at 50 nM and 100 nM of siRNA, with around 50 % inhibition of PD-L1 observed. Overall, PEG_12_-KL4 was more effective than Lipofectamine 2000 in mediating siRNA knock-down of EGFR as well as PD-L1 on NSCLC cells.

### 3.3 Cellular uptake of siRNA

**Fig. 4:**
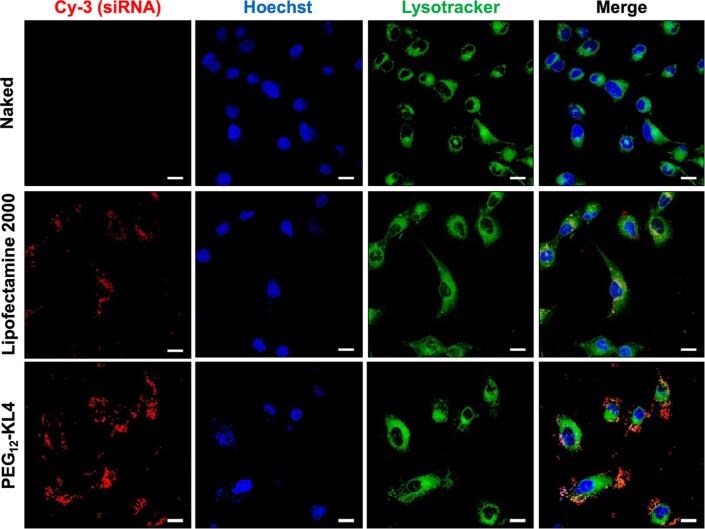
Cellular uptake of fluorescent siRNA on NCI-H1975 cells using confocal microscopy. The cells were transfected with naked siRNA, Lipofectamine 2000/siRNA complexes prepared at 2:1 ratio (w/w) and PEG_12_-KL4/siRNA complexes prepared at 10:1 ratio (w/w), all containing 2 µg of cyanine 3-labelled siRNA. Confocal images of cells transfected with cyanine 3-labelled siRNA (red); the nuclei (blue) were stained with Hoechst; the lysosomes (green) were stained with Lysotracker. Images were taken at 4 h post-transfection. Scale bars are 20 μm. (For interpretation of the references to colour in this figure legend, the reader is referred to the web version of this article.)

The transfection study suggested that PEG_12_-KL4 was superior to Lipofectamine 2000 in delivering siRNA into cells, and the effect was pronounced on NCI-H1975 cells. Live cell confocal fluorescence microscopy imaging was performed to further compare the cellular uptake efficiency of the two delivery agents on NCI-H1975 cells which were transfected with fluorescently labelled siRNA (Fig. 4). While both Lipofectamine 2000 and PEG_12_-KL4 were able to mediate cellular uptake of siRNA, a higher level of siRNA was observed in cells transfected with PEG_12_-KL4, demonstrated by the significantly higher fluorescence intensity (Fig. S1, Supplementary Information) as an indication of the fluorescent siRNA, in the confocal images. Furthermore, the locations of siRNAs in the cells transfected with PEG_12_-KL4 were distinct from the lysosomes as labelled by the lysotracker, suggesting that siRNAs were either able to escape from lysosomes after entering the cells, or they successfully bypassed the lysosomes in the cellular uptake mechanism. In contrast, co-localisation of siRNA and lysosomes were observed in cells transfected with Lipofectamine 2000, which may lead to lysosomal degradation of siRNA. This could explain the relatively poor siRNA transfection efficiency and hence the inferior gene silencing effect of Lipofectamine 2000 as compared to PEG_12_-KL4 peptide on this cell line.

### 3.4 Dual transfection of siRNA targeting both EGFR and PD-L1

**Fig. 5:**
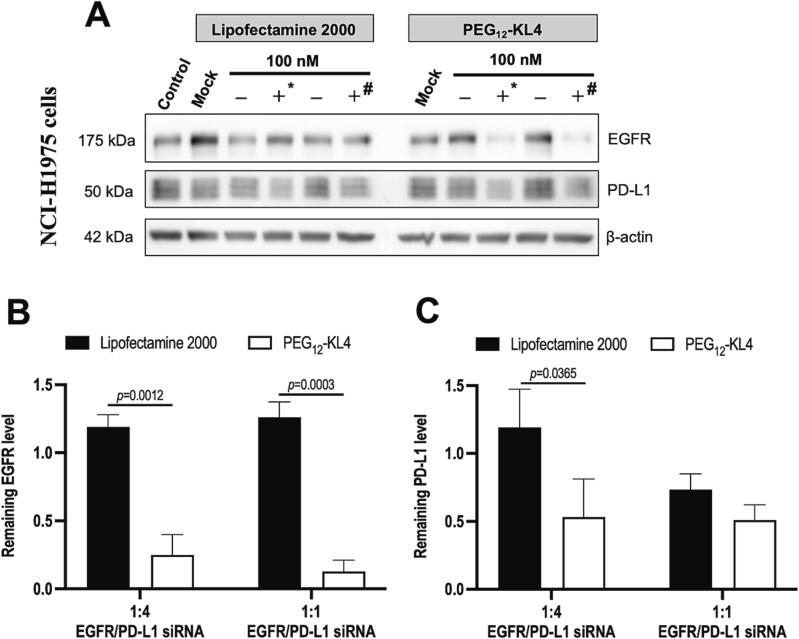
Co-transfection of siRNA targeting EGFR and PD-L1. NCI-H1975 cells were transfected with PD-L1 and EGFR siRNAs (+) or negative control siRNA (-) at a total siRNA concentration of 100 nM, where * represented 20 nM EGFR siRNA and 80 nM PD-L1 siRNA (1:4 EGFR/PD-L1 siRNA ratio); # represented 50 nM EGFR siRNA and 50 nM PD-L1 siRNA (1:1 EGFR/PD-L1 siRNA ratio). (A) Representative Western blot of EGFR and PD-L1 expression at 48 h post-transfection with β-actin as internal control. Densitometry analyses of (B) EGFR and (C) PD-L1 expressions were illustrated as the mean ± SD of three independent replicates (n = 3). The data were analysed by two-way ANOVA followed by Tukey’s post-hoc test.

As demonstrated in the previous section, PEG_12_-KL4 peptide was highly effective in transfecting siRNA on NCI-H1975 cells. Here, the ability of the peptide in delivering multiple sequences of siRNAs to inhibit EGFR and PD-L1 simultaneously was also explored on this cell line (Fig. 5). Since the knock-down efficiency was more prominent with EGFR than PD-L1, in addition to the equal split of 1:1 ratio (EGFR siRNA to PD-L1 siRNA), the 1:4 ratio was also explored with the attempt to maximise the inhibition of both proteins without excessive high dose of siRNA. The total combined siRNA concentration was 100 nM in both ratios. The expressions of both EGFR and PD-L1 were significantly reduced by dual siRNA transfection, and the delivery efficiency of PEG_12_-KL4 was remarkable. The levels of EGFR and PD-L1 expression were reduced to around 20 % and 50 %, respectively. However, there was no significant difference between the two ratios of siRNAs. Consistent with the cellular uptake study, PEG_12_-KL4 was more efficient in mediating siRNA knock-down on NCI-H1975 cells than Lipofectamine 2000, possibly due to the more favourable uptake pathway that allows the siRNA to either escape or bypass lysosomal degradation.

The expression of PD-L1 was associated with EGFR mutation [25]. Evidence of other studies suggested that inhibition of EGFR could modulate the tumour immune environment, which could sensitise cancer cells to immunotherapy and increase the proportion of patients benefit from PD-1/PD-L1 immune checkpoint inhibitors [26], [27]. While combination therapy by using EGFR-TKIs with PD-L1 monoclonal antibodies can enhance and prolong treatment efficacy, as well as prevent or delay the occurrence of more than one resistance mechanism [28], [29], [30], there has been concerns with the overlapping toxicities when delivering both agents together [14], [31]. For instances, the combined application of osimertinib (third generation EGFR-TKI) and durvalumab (anti-PD-L1 monoclonal antibody) had an encouraging objective response rate of 70 % in NSCLC patients harbouring EGFR mutation [32]. However, the combination approach was also associated with high incidence of interstitial lung disease which often led to progressive scarring of lung tissue. The damage was generally irreversible, and the mechanism of toxicity is poorly understood [14], [33]. Current data with promising dual inhibition of EGFR and PD-L1 by siRNAs might be an alternative approach to overcome the limitations of combinational therapy.

### 3.5 Cytotoxic activity of CD8+ T cells in NSCLC cells transfected with EGFR and PD-L1 siRNAs

**Fig. 6:**
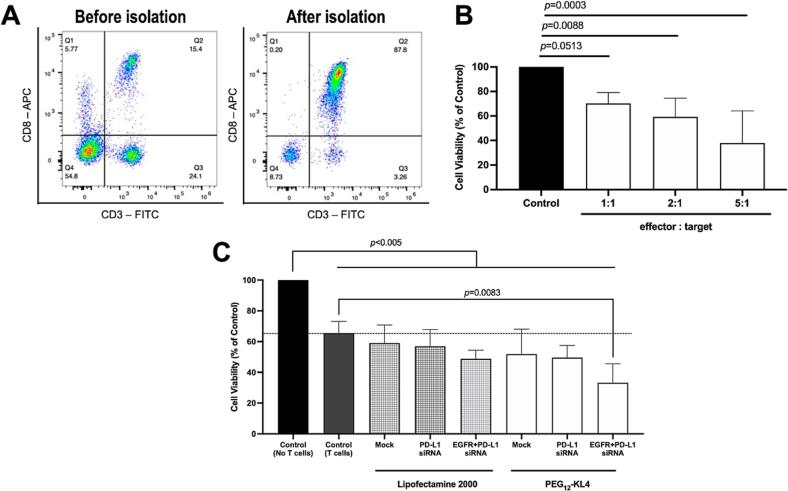
Co-culture of isolated human CD8+ T cells with NSCLC cells. (A) Human PBMCs (before isolation) and CD8+ T cells (after isolation) were stained with anti-human CD3 antibody-FITC and anti-human CD8a antibody-APC. The cells were analysed by a flow cytometer to quantify the FITC and APC fluorescence intensity. (B) NCI-H1975 cells were co-cultured with CD8+ T cells in a range of effector: target ratios for 24 h. The cell viability was measured by MTT assay. (C) NCI-H1975 cells were transfected with siRNAs targeting PD-L1 alone or both EGFR and PD-L1 (at 1:1 ratio), using either Lipofectamine 2000 or PEG_12_-KL4 as the transfection agent for 48 h. CD8+ T cells were added to co-culture with NCI-H1975 cells at effector: target ratio of 2:1 for additional 24 h. The cell viability was evaluated by MTT assay to examine T cell killing activity. The data was presented as the mean ± SD of three independent replicates (n = 3). The data were analysed by one-way ANOVA followed by Tukey’s post-hoc test.

The rationale of inhibiting PD-L1 expression on cancer cells was to reduce their ability to interact with PD-1 on T cells, thus to limit this inhibitory axis and resulting in more T cell-mediated cancer cell killing when bringing both cell population into contact [34]. We examined the efficiency of primary human CD8+ T cells to kill NCI-H1975 cells that had been treated with either control siRNA or PD-L1-targeting siRNA. Human CD8+ T cells were enriched from peripheral blood mononuclear cells obtained from healthy donors (15.4 % enriched to 87.8 %; Fig. 6A). Such enriched CD8+ T cells were co-cultured with NCI-H1975 cells at effector: target ratios of 1:1, 2:1, and 5:1. The cytotoxic activity of CD8+ T cells significantly increased with higher effector: target ratios, as reflected by a gradual reduction in cancer cell viability (Fig. 6B). The effector: target ratio 2:1 was selected for subsequent experiments as it represented a good compromise (∼60 % of NCI-H1975 survival enabling assessment of any added cytotoxic effects caused by PD-L1 knock-down).

When NCI-H1975 cells were transfected with siRNAs targeting both EGFR and PD-L1 mediated by PEG_12_-KL4, the cell viability was significantly reduced after co-culturing with CD8+ T cells for 24 h (Fig. 6C). The cell viability reduced from around 60 % in cells without siRNA transfection, to around 30 % in cells transfected with both EGFR and PD-L1 siRNAs. The cell viability in this treatment group was the lowest among all the treatment groups, indicating that the knock-down of both EGFR and PD-L1 protein expression efficiently enhanced the cytotoxic function of CD8+ T cells. Interestingly, single knock-down of PD-L1 was insufficient to improve CD8+ T cell killing, which suggested that dual inhibition of EGFR and PD-L1 could be a better strategy than just knocking down PD-L1 alone. In fact, dual targeting EGFR and PD-L1 may display certain synergistic effects. Previous studies showed evidence that targeted therapies using EGFR-TKIs alleviate tumour-mediated immunosuppression via the suppression of tumorigenic inflammation and immunosuppressive cells such as myeloid- derived suppressive cells, tumour-associated macrophages, tumour-associated neutrophils, and regulatory T cells (Tregs) [35], [36]. Their findings suggest that EGFR-TKIs could enhance the infiltration of immune cells into the tumour, strengthening the effectiveness of immunotherapy. Furthermore, another preclinical study demonstrated that EGFR-TKIs indirectly enhanced the anti-tumour activity via inhibiting the functions of immunosuppressive Tregs through the EGFR/GSK-3/FOXP3 axis [37] and educating the tumour-specific T cells to release inflammatory cytokines such as IFN-γ, which in turn recruited the peripheral natural killer (NK) cells to attack the tumour [20]. Targeting EGFR may help to modulate tumour microenvironment, that is favourable to the potency of immunotherapeutic. Therefore, combination of both therapies is feasible to enhance the anti-tumour immunity, potentially leads to an overall improved patient outcome. A recent study reported the development of a bispecific antibody that targeted both PD-L1 and EGFR with promising results against breast and lung cancer in animal models [38]. Further investigation on the dual siRNA targeting approach is warranted.

### 3.6 *In vivo* biodistribution of fluorescent siRNA

**Fig. 7:**
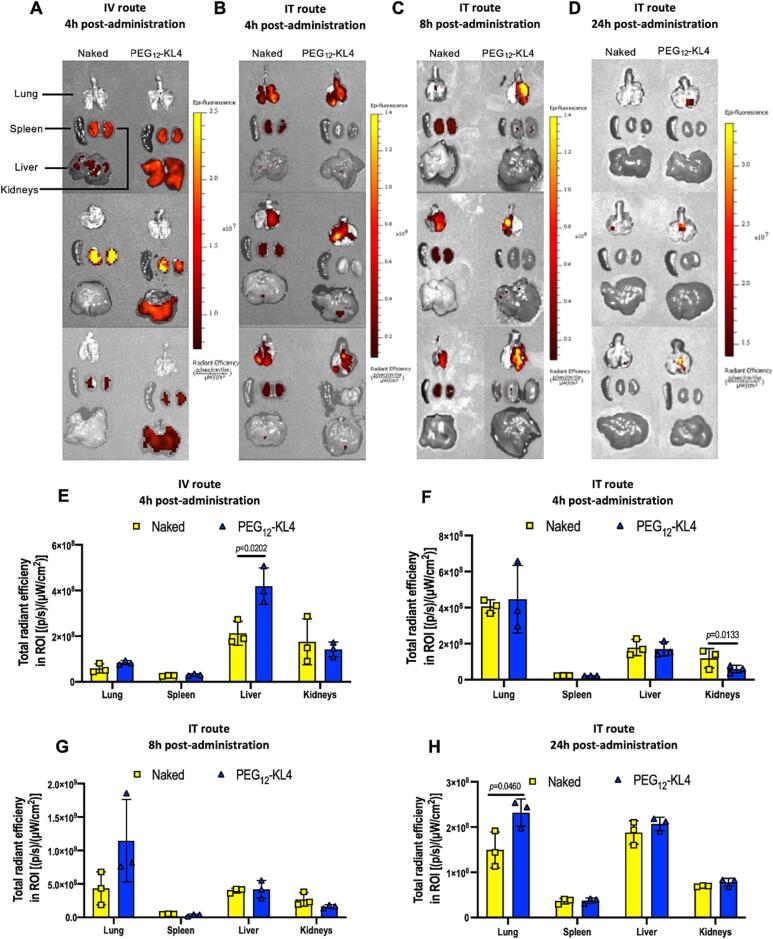
Biodistribution of fluorescently labelled siRNA following intravenous (IV) or intratracheal (IT) administration in mice. BALB/c mice were administered with either naked siRNA or PEG_12_-KL4/siRNA complexes. (A-D) The lungs, liver, kidneys, and spleen were isolated and imaged. (E-H) The fluorescence signals were measured at (A, E) 4 h after IV administration; (B, F) 4 h after IT administration; (C, G) 8 h after IT administration; and (D, H) 24 h after IT administration. The data was presented as the mean ± SD (n = 3). The data were analysed by Student’s *t*-test to compare between the biodistribution of fluorescent siRNAs in indicated organs with or without PEG_12_-KL4.

Poor *in vivo* delivery efficiency remains a major hurdle for the translation of siRNA therapeutics in clinical use for lung diseases [39]. To date, all approved siRNA therapeutics are administered parenterally, which is suboptimal for the management of lung conditions. For lung cancer treatment, pulmonary delivery could offer an effective approach to localise siRNA in the lung tissues and thereby allow effective gene silencing at the tumour sites while minimising side effects [40], [41], [42]. So far, PEG_12_-KL4 has demonstrated its ability to mediate effective siRNA induced knock-down of EGFR and PD-L1 on NSCLC cell lines. Here, we investigated the biodistribution of fluorescent siRNA following intratracheal administration and compared it with the intravenous route of administration (Fig. 7).

Following intravenous administration, fluorescent siRNA was found to be accumulated in the liver in the cohort receiving PEG_12_-KL4/siRNA complexes (Fig. 7A and E), and the level of liver fluorescence was significantly higher than in the cohort receiving naked siRNA via the same administration route. siRNA could also be detected in the kidneys in both cohorts, suggesting siRNA elimination through the renal route following intravenous administration. Notably, there was hardly any siRNA detected in the lung. These results indicated that while PEG_12_-KL4 could be useful in delivering siRNA to the liver by intravenous administration, as seen in many other nanoparticulate delivery systems [43], this route of administration was ineffective in delivering siRNA to the lung, at least with PEG_12_-KL4. On the other hand, administration of PEG_12_-KL4/siRNA complexes through the pulmonary route led to the accumulation of siRNA in the lungs as expected (Fig. 7B and F). While both cohorts that received naked siRNA and PEG_12_-KL4/siRNA complexes showed similar levels of siRNA in the lungs 4 h after administration, the PEG_12_-KL4/siRNA cohorts showed higher levels of fluorescence signals 8 h and 24 h after administration (Fig. 7C, D, G and H). This demonstrated the capability of PEG_12_-KL4 to prolong siRNA residence time and/or stability in the lung. In line with this observation, the fluorescent siRNA levels in the kidneys of the cohort receiving naked siRNA was significantly higher than those in the cohort receiving PEG_12_KL4/siRNA complexes 4 h after administration, suggesting faster renal excretion of the naked siRNA.

Our biodistribution data suggested that the PEG_12_-KL4 peptide featured desirable characteristics that allow siRNAs to be accumulated in the lung following pulmonary delivery. A number of studies also reported the pulmonary delivery of siRNA, using lipid- or polymer-based nanoparticles as delivery vectors, to inhibit targets such as Kirsten rat sarcoma viral oncogene homologue (KRAS) protein, βIII-tubulin chemokine receptor CXCR4 and activator of transcription 3 (STAT3) as potential treatment of NSCLC or lung metastases [44], [45], [46]. Regardless of the types of delivery system employed, it was often noticed that pulmonary administration resulted in better lung accumulation as compared to the intravenous route [44], [45], [47]. Taken together, pulmonary siRNA delivery is a non-invasive locally targeted administration route that could offer more efficient uptake by the cancer cells in the lung, and hence deserves further investigation.

## 4 Conclusions

This work demonstrated the utility of the synthetic PEG_12_-KL4 peptide to deliver siRNAs targeting both EGFR and PD-L1 simultaneously as a potential therapeutic strategy against NSCLC. PEG_12_-KL4 was shown to be an excellent siRNA transfection agent in various NSCLC cells to mediate effective silencing of both EGFR and PD-L1. The success of PEG_12_-KL4 in transfecting siRNA was at least in part attributed to its ability to bypass or escape lysosomal degradation after cellular uptake. T cell killing assays revealed that co-delivery of siRNA targeting both EGFR and PD-L1 by the PEG_12_-KL4 peptide significantly enhanced the cytotoxic effects driven by CD8+ T cells, thereby illustrating the feasibility of this dual silencing of EGFR and PD-L1 approach against NSCLC. Finally, we demonstrated through *in vivo* biodistribution experiments that the pulmonary route is a better route of administration to the lung than intravenous delivery in this context with the use of PEG_12_-KL4 peptide. Future studies will focus on the antitumour effects of this delivery strategy in animal lung cancer models.

## CRediT authorship contribution statement

**Rico C.H. Man:** Data curation, Formal analysis, Investigation, Methodology, Writing – original draft, Writing – review & editing. **Yingshan Qiu:** Data curation, Investigation, Methodology. **Susan W.S. Leung:** Resources, Supervision, Writing – review & editing. **Gilbert O. Fruhwirth:** Conceptualization, Formal analysis, Funding acquisition, Project administration, Resources, Supervision, Validation, Writing – review & editing. **Jenny K.W. Lam:** Conceptualization, Funding acquisition, Project administration, Supervision, Writing – original draft, Writing – review & editing, Resources.

## Data Availability

Data will be made available on request.
