# Supplementary material for: Co-delivery of PD-L1- and EGFR-targeting siRNAs by synthetic PEG12-KL4 peptide to the lungs as potential strategy against non-small cell lung cancer
Source: Eur J Pharm Biopharm. 2024 Feb;195:None. doi: 10.1016/j.ejpb.2024.114177 (PMC11932971; doi:10.1016/j.ejpb.2024.114177)
Supplement: Supplementary data 1 [file mmc1.docx]

**Supplementary Information**

**Table S1.1.** Information siRNA targeting EGFR used in the transfection study. A pool of siRNAs consisting of four individual sequences at equal ratio were used, and the siRNA sequences are shown.

| **EGFR siRNA** | **Sequence** |
| --- | --- |
| siRNA 486 | Sense (5’→3’) GAC CAU CCA GGA GGU GGC UGG UUA UUU  Anti-sense (5’→3’) AUA ACC AGC CAC CUC CUG GAU GGU CUU |
| siRNA 604 | Sense (5’→3’) GCA GUC UUA UCU AAC UAU GAU GCA AUU  Anti-sense (5’→3’) UUG CAU CAU AGU UAG AUA AGA CUG CUU |
| siRNA 752 | Sense (5’→3’) GCA GUG ACU UUC UCA GCA AUU  Anti-sense (5’→3’) UUG CUG AGA AAG UCA CUG CUU |
| siRNA 1247 | Sense (5’→3’) GCA AAG UGU GUA ACG GAA UAG GUA UUU  Anti-sense (5’→3’) AUA CCU AUU CCG UUA CAC ACU UUG CUU |

**Table S1.2.** Information of siRNA targeting PD-L1 used in the transfection study. A pool of siRNAs consisting of four individual sequences were used. The target sequences are shown below, but the siRNA sequences and the ratios between each sequence were not provided by the manufacturer.

| **PD-L1 siRNA** | | **Target sequence** |
| --- | --- | --- |
| ON-TARGETplus Human PD-L1 siRNA | siRNA J-015836-09 | GCC GAC UAC AAG CGA AUU A |
|  | siRNA J-015836-10 | GGC AUU UGC UGA ACG CAU U |
|  | siRNA J-015836-11 | GAA AAU GGA ACC UGG CGA A |
|  | siRNA J-015836-12 | CCU AUA UGU GGU AGA GUA U |

**Table S1.3.** Information of fluorescently labelled siRNA used in the cellular uptake study. The target sequence is shown below, but the siRNA sequence was not provided by the manufacturer.

| **Fluorescently labelled siRNA** | | **Target sequence** |
| --- | --- | --- |
| siGLO Cyclophilin B Control siRNA | D-001610-01-50 | GGA AAG ACU GUU CCA AAA A |

**Fig. S1. Cumulative quantitative intensity analysis of cellular uptake of fluorescently labelled siRNA on NCI-H1975 cells.** The cyanine 3 fluorescence signals, which were indicative of siRNA, were quantified. The data were presented as the mean ± SD of three independent images (n=3). The data were analysed by one-way ANOVA followed by Tukey’s multiple comparison test.
